# Vocal functional flexibility in the grunts of young chimpanzees

**DOI:** 10.1016/j.isci.2023.107791

**Published:** 2023-08-30

**Authors:** Derry Taylor, Erik Gustafsson, Guillaume Dezecache, Marina Davila-Ross

**Affiliations:** 1 University of Portsmouth, Psychology Department, King Henry Building, Portsmouth PO1 2DY, UK; 2 University of Neuchâtel, Institute of Biology, Department of Comparative Cognition, Rue Emile-Argand 11, 2000 Neuchâtel, Switzerland; 3 Université Clermont Auvergne LAPSCO CNRS, Bâtiment Paul Collomp, TSA 60401, 34, Avenue Carnot, 63037 Clermont-Ferrand, France

**Keywords:** Zoology, Anthropology, Linguistics

## Abstract

All living things communicate yet only humans can be said to communicate using language. How this came to be the case is a fundamental mystery unsolved by contemporary science. Within a human lifetime, language emerges from a complex developmental process. As such, understanding chimpanzee vocal development is essential to understanding the evolutionary roots of language. In human development, language is directly built upon the early capacity for “vocal functional flexibility”—the ability to flexibly express the same vocalizations in different ways to achieve different functions. Primate vocalizations, by contrast, have long been believed to be relatively inflexible regarding both production and function. In this paper, we break new ground by providing evidence for vocal functional flexibility in one of the first systematic studies of early chimpanzee vocal production and function. This finding implies the developmental foundations for language are rooted in our primate evolutionary heritage.

## Highlights

• Most immature chimpanzee call types are stereotyped in how they are produced
• Grunts were flexibly expressed and showed a corresponding flexibility in function
• A key developmental foundation for language appears rooted in our primate ancestry

## Graphical abstract

**Figure:**
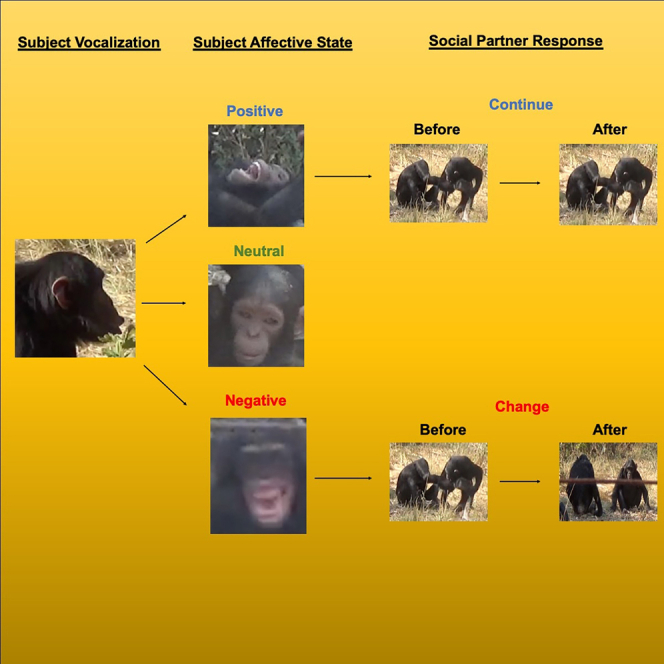

## Introduction

Human language is uniquely versatile and highly effective.^1^^,^^2^ The same linguistic utterance (i.e., the same sequence of words) can be flexibly produced to express different meanings which achieve different functions on different occasions.^3^^,^^4^ The mere presence of cultural variants is impossible without this characteristic. As such, language is marked by a decoupling of signal form and function, such that the same signal (e.g., “the train arrives”) can be used to express frustration (meaning: the train *finally* arrives), joy (meaning: the train arrives and I will finally be able to see my spouse), or a neutral stance (meaning: the train arrives’ uttered by the station speaker) on different occasions.

This ability to achieve different functions by expressing the same utterance in different ways is known as “functional flexibility.”^3^ Specifically, “functional flexibility” requires the capacity to (i) express a call with different illocutionary forces corresponding to what the call conveys (criterion 1) and (ii) to elicit a consistent response in the receiver depending on the context in which the call is expressed (criterion 2). Functional flexibility is evident from early in human development. Among 3–12-month-old human infants, Oller and colleagues^3^ found protophones, which are believed to be precursors to speech sounds,^4^^,^^5^ express positive, neutral, and negative affective states on different occasions based on behavioral indicators of affect that co-occurred with the vocalizations (criterion 1). Also, when human infant protophones expressed positive affect, caregivers mostly responded with attempts to “encourage” continuation of social interactions, whereas negative protophones were associated with attempts to “change” the infants’ state (criterion 2). This was not the case for laughs and cries. Crucially, this demonstrates flexibility in protophone production provides a foundation for communication that is meaningful in some sense and may therefore plausibly provide a foundation for language.

While vocal functional flexibility seems to have deep biological roots in humans as indicated by its presence from the first months of human ontogeny, and has received much research attention from an evolutionary perspective,^6^^,^^7^^,^^8^ it has much been reported that signal form and function are tightly coupled in non-human primate calls.^1^^,^^9^^,^^10^ For instance, it has been reported that chimpanzee vocalizations are often closely tied to the experience of a given and specific emotion.^11^^,^^12^ However, there is growing research suggesting some degree of flexibility in primate vocalizations regarding both contextual production and call acoustics.^2^^,^^13^^,^^14^^,^^15^

Furthermore, there are two studies that were designed to test for vocal functional flexibility in Pan. Specifically, a study on adult bonobo peep calls^16^ and another on infant chimpanzee grunts and whimpers^17^ examined the criterion 1 of functional flexibility closely. In both studies, the authors classified vocalizations as positive, neutral, or negative and found calls (only infant grunts in the study by Dezecache et al^17^) were produced across all affective 3 states. It is, however, important to note that the classification of the vocalizations was done based on overall behavioral contexts for the bonobos.^16^ In contrast, the classification was based on the behavioral cues of the subjects for the chimpanzees^17^ matching the approach used for human infants. This research provided evidence for criterion 1 in Pan. Crucially, this shows that beyond merely flexible vocal production across contexts, in which case calls may communicate the same information that happens to be relevant in a wide range of contexts^15^ the calls of these studies communicated different messages on different occasions (i.e., affective states). Although, social partner responses were not assessed, meaning these studies did not provide evidence that these vocalizations serve different functions, i.e., criterion 2. Consequently, there is the need to test if functional flexibility is present in animal calls. This represents a critical gap in our understanding of the evolutionary origins of language.

Communicative ontogeny more generally is heavily understudied in primatology. In chimpanzee vocal communication, studies were mostly descriptive and based on relatively small sample sizes.^18^ More recent systematic and quantitative studies have shown that while gesturing peaks in infancy and decreases thereon, vocal behavior increases.^10^ During vocal ontogeny, it has also been recently shown that vocal behavior becomes more acoustically complex.^8^ However, aside from a recent study of pant hoots observed in a single infant chimpanzee,^19^ systematic studies of the vocal behavior of young chimpanzees are almost entirely absent. Yet systematic studies of early human vocal behavior have shown that the foundations for complex communication are laid within the first few months of ontogeny.^1^^,^^4^

Given the importance of studying early vocal behavior in humans for understanding the roots of language^1^^,^^3^^,^^4^ adopting a comparative-developmental approach in the present study we aimed to test for vocal functional flexibility in young chimpanzees by following the protocol used to study functional flexibility in human infants.^3^ We examined 28 infant and juvenile chimpanzees from four semi-wild colonies at Chimfunshi Wildlife Orphanage, Zambia, focusing on the most commonly produced calls that immature chimpanzees produce, i.e., grunts, whimpers, laughs, screams, and hoo calls.^8^ To test for criterion 1, we classified subjects’ affective states as positive, neutral, or negative using facial expressions and bodily actions and examined whether each call type was significantly biased toward expressing particular affective states. To test for criterion 2, we examined whether social partners changed or continued their behaviors depending on the affective valence of a call.

## Results

We examined 768 calls in total (average of 27.8 per individual). The call types included grunts (N = 382), whimpers (N = 147), laughter (N = 139), screams (N = 41), hoo calls (N = 41), barks (N = 8), squeaks (N = 6), and pant hoots (N = 4).

### Criterion 1

**Figure 1:**
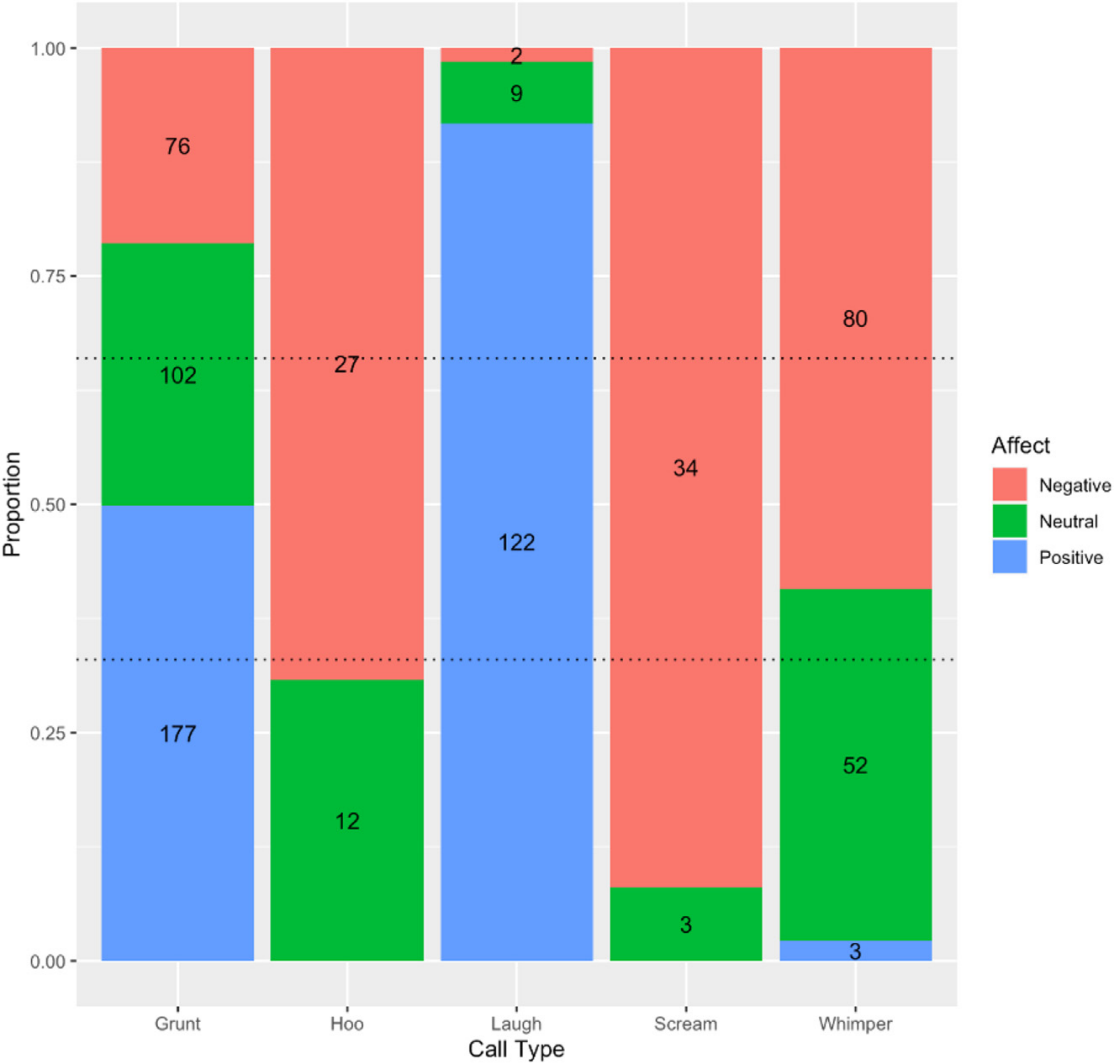
Stacked bar chart showing the proportion of calls produced across positive, neutral, and negative affective states for each call type based on the total number of events Dashed lines indicate expected proportions assuming a random distribution across affective states. Raw number of calls per category are overlaid on the bars.

Distribution of call production across affective states is shown in Figure 1. To test for the extent to which criterion 1 is satisfied in the vocal repertoire of young chimpanzees (i.e., the degree of affective bias in vocal expression), we first calculated a “Berger-Parker diversity index” value^17^^,^^20^ per call type for each subject. To obtain the value, we divided the number of times a call type was produced in the most common affective state by the total number of calls produced across all affective states. Berger-Parker diversity index values range from 0.33 (1 divided by the number of affective states, in this case 1/3), indicating no bias, up to 1, indicating complete affective bias. One-sample two-tailed Wilcoxon tests revealed that affective bias in grunts and whimpers was significantly lower than the hypothetical median Berger-Parker diversity index value of 1 (grunts: V = 0, p = 0.0004; whimpers: V = 0, p = 0.0006). By contrast, laughs (V = 0, p = 0.059), screams (V = 0, p = 0.371), and hoo calls (V = 0, p = 0.371) were not found to be significantly less biased.

There were also no significant differences between infants and juveniles in affective bias within call types (grunts: W = 34.5, p = 0.310; whimpers: W = 44, p = 0.782; laughs: W = 36, p = 1.000; screams: W = 30, p = 0.244). Test statistics could not be calculated for an infant-juvenile comparison of hoo call production due to a small number of infants producing hoo calls (N = 3). Together, these results suggest that in infants and juveniles, both grunts and whimpers are produced flexibly across affective states, whereas screams, hoo calls, and laughs show a strong affective bias.

### Criterion 2

**Figure 2:**
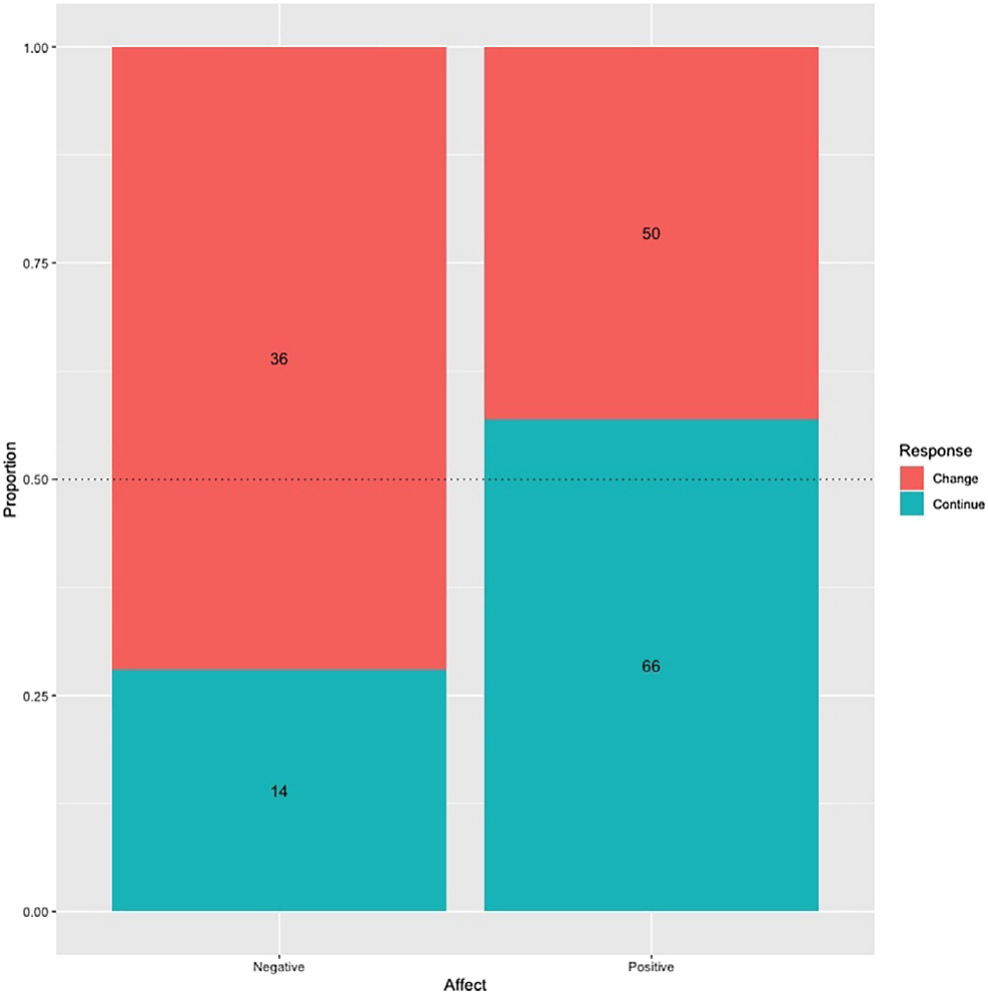
Stacked bar chart showing the contingency between vocal affect (positive vs. negative) and social partner responses (change vs. continue) for grunts The dashed line indicates expected proportions assuming a random distribution of responses. Raw number of responses per category are overlaid on the bars.

Among all call types only grunts and whimpers satisfied criterion 1. However, for whimpers, only 3 calls were observed to express positive affect. This was insufficient for statistical analysis, and we therefore limit our analysis of criterion 2 (i.e., whether social partners showed consistent behavioral responses to vocalizations depending on how those calls were expressed) to grunts. We used McNemar tests in order to evaluate whether there was a significant statistical dependency between the affective state a call expressed (i.e., positive vs. negative) and the social partner response (i.e., continue vs. change behavior, respectively, see Table 1 for coded social partner behaviours). These data are shown in Figure 2. A McNemar test comparing the relationship between grunt affect (positive vs. negative) and social partner response (change vs. continue) showed that negative grunts were more likely to elicit behavioral change in a social partner while positive grunts were more likely to elicit behavioral continuation (χ^2^ = 8.24, p = 0.002). 72% of negative grunts elicited behavioral change and 28% elicited behavioral continuation. 56% of positive grunts elicited behavioral continuation and 44% elicited behavioral change. Further details on the behavioral responses of mothers and non-maternal social partners in relation to subject behavior and facial expression during grunt production are shown in Tables S5 and S6 respectively.

**Table 1:** Categories of social partner behavior directed toward subject and behaviors that belong to each category

Category	Behavior directed to subject
Feeding offspring	Food sharing, breastfeeding
Protecting	Defending, gathering subject
Comforting	Cradling, patting, embracing, bite-kissing, holding hand, extending hand
Playing	Play wrestling, tickling
Grooming	Grooming, inspecting
Approach	Approaching subject
Travel	Lowering back for subject to climb on, following, carrying subject
Avoidance	Avoiding, leaving, or breaking contact outside of play
Preventing breastfeeding	Covering nipple (mothers only)
Threat	Arm raising gesture, biting at (outside of play), dominance displays
Taking	Taking or pulling objects in subjects’ possession
Causing discomfort	Pulling, slapping, hitting, dragging, pulling hair, pushing (outside of play).

See^28^ for definitions.

## Discussion

The present study aimed to test for functional flexibility in the vocal production of young chimpanzees. It was found that for both infants and juveniles, the vocal repertoire comprises a mixture of flexibly expressed and more affectively biased call types, much like human infants. We also found evidence for functional flexibility among flexibly expressed call types, with grunts systematically eliciting different responses from social partners depending on how they were expressed with co-occurring behaviors. Together, these results suggest that functional flexibility features in vocal production among young chimpanzees.

Our findings show a clear parallel with human infant research. In human infant research^3^ it has been shown that laughs and cries are more affectively biased than protophones, which in turn are also more functionally flexible. Here, flexibly expressed vocal units were also more functionally flexible—social partner responses to grunts were dependent on the affective state the call expressed on a given occasion. This is the first study to show a non-human primate call type appears to be functionally flexible in this sense. This helps to explain why flexibly expressed signals might be expected to be present in non-human primates, because if signals do not systematically affect receivers they are unlikely to be selected for.^21^ Therefore, flexible vocal expressivity could plausibly offer a selective benefit. It is important to appreciate the apparent differences between human infants and young chimpanzees also. Namely, that human infant vocalizations are often produced when there is no interaction with social partners,^3^ whereas young chimpanzee vocalizations in this study were almost always tied to social interactions.

Importantly, in a recent study it was demonstrated that an unsupervised machine learning algorithm spontaneously classified each call type in this study as belonging to distinct call classes that correspond with the original behavioral coding^8^ suggesting that grunts are indeed a single call class expressed differently on different occasions. Further, given the range of behavioral indicators of affect used, it is unlikely that our findings can simply be explained as multi-modal signal combinations with systematic functions that differ to the functions of single elements of those combinations. Since functional flexibility is believed to be essential for language emergence^1^^,^^3^ our findings could suggest that language emerged in the hominin lineage because the essential pre-requisites were already present in that lineage.

To conclude, functional flexibility appears to be present in the vocal communication of immature chimpanzees. In human development, language is indeed built upon a pragmatic foundation of flexible expressivity and functional flexibility. The present study contradicts the long-held view of primate vocal production as a rigid modality and adds to a growing body of literature showing flexibility in primate vocal production,^2^^,^^13^^,^^14^^,^^15^ suggesting that language may be built upon this foundation not only in development, but also in evolution. Although, it is notable that for the most part, production and function are closely tied in the chimpanzee vocal repertoire, whereas signal-function decoupling may be a more general feature of vocal communication among adult humans.

### Limitations of the study

The present findings on patterns of vocal production and function in immature chimpanzees may be limited by the system used for classifying affective states, because it is inevitably vulnerable to misclassification. Behavioral cues were central to affect classification in the present study. While many believe behavioral cues can communicate affective information,^22^ an alternative view is that such behaviors are “action-intention” cues—cues that provide others with information about what an individual is likely to do next.^23^^,^^24^ On the action-intention view, behavioral cues are not necessarily indicative of any underlying affective state. This may be seen to question whether the present coding scheme classified affective state rather than another construct such as action-intentions, which would in turn question whether our data really show functional flexibility (i.e., flexible expression of *affect* and a corresponding flexibility in function). We make several arguments against this interpretation. Firstly, we do not see that these are mutually exclusive possibilities (i.e., a cue might indicate what an individual is likely to do because of the affective state associated with it). Secondly, a variety of sources of empirical evidence do show that such behavioral cues are often associated with different affective states (see Table 2). Therefore, while behavioral cues may not always be underpinned by affective states, they often are. As such, the present coding scheme is not considered to be a perfect system for classifying affective states, but we do argue it represents an improvement upon previous attempts that relied on a much smaller range of behaviors and did not include facial expressions^16^^,^^17^ which are among the most extensively studied and reliable affective cues.^43^ Importantly, chimpanzee facial expressions and bodily behaviors can be produced independently of vocalizations^26^^,^^34^ and therefore provide independent evidence of affective state. Finally, it is important to be clear that the question at hand here is whether immature chimpanzees express different affective states, rather than whether those underlying affective states are truly occurring in a particular case. As such, we believe the present coding scheme was sufficient to provide reliable insights into affective expression of chimpanzee calls.

**Table 2:** Definitions of facial expressions, bodily actions, and associated valence with supporting literature

Behavior	Definition	Valence
**Facial Expressions**		

Full open-mouth face	An expression of continuously changing movements with cheeks and upper lips raised, lip corners pulled pack, and lower lip depressed while the jaw is lowered so the mouth is open. Bottom and top teeth visible.	Positive^25^^,^^29^
Half open-mouth face	Identical to the full open-mouth face, but with only the bottom teeth visible.	Positive^29^
Open-mouth bared-teeth	A rigid expression with cheeks and upper lips raised, lip corners pulled pack, and lower lip depressed while the jaw is lowered so the mouth is open. Bottom and top teeth visible.	Negative^25^^,^^27^^,^^30^
Closed-mouth bared-teeth	Upper lip raised, lips corners pulled back, and lower lip depressed. Upper and lower teeth usually visible	Negative^27^^,^^30^
Pout	Lips separated and funneled outwards.	Negative^11^^,^^31^^,^^32^
No Expression	Absence of any coherent facial muscle activations recognized as an expression in previous literature.	Neutral^32^^,^^33^

**Bodily Actions**		

Play actions	Play wrestling, pirouetting, solitary play, and somersaulting (see Plooij, 1984)^28^	Positive^34^^,^^35^
Grooming actions	Picking through the fur of another individual.	Positive^36^
Breastfeeding	Having mother’s nipple in mouth. Actual sucking movements may or may not be seen.	Positive^37^
Nuzzling	Unsuccessfully attempting to access the mother’s nipple.	Negative^11^
Aggressive actions	Tantrums, hitting, slapping, pulling hair outside of playing, and dominance displays (see Plooij, 1984).^28^	Negative^11^^,^^35^
Self-scratching	Moving the nails over the skin of some part of the own body while bending the fingers.	Negative^31^^,^^38^^,^^39^
Avoidance actions	Avoiding approaching conspecifics, resisting physical contact of conspecifics, and defending objects from conspecifics (see Plooij, 1984).^28^ All behaviors must occur outside of play.	Negative^40^
Other actions	All coded behaviors that were not included in the above were considered to be neutral. Common examples are locomotion, climbing, laying down, sitting, object manipulation, and traveling.	Neutral^41^^,^^42^

## STAR★Methods

### Key resources table

**Table:** 

REAGENT or RESOURCE	SOURCE	IDENTIFIER
**Experimental models: Organisms/strains**		

Pan troglodytes	Chimfunshi Wildlife Orphanage, Zambia	N/A

**Deposited data**		

Original data deposited for this study	Supplementary materials of this article	N/A

**Software and algorithms**		

Code for data analysis	Supplementary materials of this article	N/A
R: A language environment for statistical computing v2023.03.0	R core team, 2022	http://www.r-project.org

### Resource availability

#### Lead contact

Further information and requests for resources and reagents should be directed to and will be fulfilled by the lead contact, Derry Taylor (derry.taylor@unine.ch).

#### Materials availability

This study did not generate new unique reagents.

### Experimental model and study participant details

Research complied with the protocols approved by the University of Portsmouth Animal Ethics Committee and the Chimfunshi Wildlife Orphanage Research Advisory Board. The data used in this study are non-invasive video recordings of 28 semi-wild chimpanzees, including 15 males and 13 females, housed at Chimfunshi Wildlife Orphanage. Given the almost even number of males and females in our study, we do not believe sex has likely impacted our results.

### Method details

#### Subjects and study site

Subjects were infant (N=15) and juvenile (N=13) semi-wild chimpanzees housed at Chimfunshi Wildlife Orphanage, Zambia. Infant ages ranged from 2 months up to 4 years of age (M=1.76 ± SD=1.14). Individuals aged between 4 and 10 years were classified as juveniles (M=6.87 ± SD=1.65). These age ranges were chosen because according to the overview of Laporte (2011), these age ranges are widely agreed upon based on distinct behavioural and morphological traits associated with each stage. All subjects were raised by their mothers during infancy. All infant subjects still lived with their biological mothers. Three juveniles did not live with their mothers due to fatalities that occurred in years prior to the present study. All subjects in the present study were born and raised in semi-wild sanctuary conditions. Each subject belongs to one of four mixed-sex colonies that comprise between 10 – 52 members. The four colonies lived in four outdoor enclosures, respectively. The enclosures range between 47 and 190 acres in size.

#### Data collection

Video and audio recordings were collected between 7am and 6pm from June 2018 to October 2018 using a Sony CX405 Handycam with a Sennheiser ME66 directional microphone attached. Recordings were collected only when the subjects were outdoors and the recordist was within 2–10 meters of the subject. The main approach in collecting recordings was to use a 5-minute focal sampling. Overall, between 15 and 51 focal recordings were collected per subject, meaning focal observation time ranged between 1.24 and 4.25 hours of recording per subject (Infants: M=2.72 ± SD=0.96; Juveniles: M=3.43 ± SD=0.04). The total duration of *ad libitum* recordings per subject ranged between 0.03 hours and 3.52 hours (Infants: M=0.81 ± SD=0.79; Juveniles: M=0.18 ± SD=0.15). The total duration of incomplete focal recordings ranged between 0.20 hours and 1.27 hours per subject (Infants: M=0.70 ± SD=0.33; Juveniles: M=0.55 ± SD=0.28). Overall, total observation time ranged between 1.73 hours and 5.45 hours per subject (Infants: M=4.19 ± SD=1.31; Juveniles: M=4.16 ± SD=0.53).

#### Identifying calls

The coded unit of vocal behaviour was the call type, which is a broad category of calls (i.e. grunts) that contains distinct variants (i.e. food grunt, pant grunt, etc). Call types were chosen because there is wide agreement regarding the call types produced by immature chimpanzees^11^^,^^28^^,^^44^ but whether immature chimpanzees exhibit distinct subtypes (i.e. food grunts, pant grunts, etc) is currently unclear due to a lack of systematic study. The relationship between call subtypes and affective state is therefore currently unknown and remains to be explored in future studies. Calls could be comprised of a single call element, or a series of call elements otherwise known as a call ‘bout’. In total, 768 calls were identified. The call types included grunts (N=382), whimpers (N=147), laughs (N=139), screams (N=41), hoo calls (N=41), barks (N=8), squeaks (N=6), and pant hoots (N=4). Calls were identified based on auditory cues followed by systematic visual inspection of spectrograms according to the definitions used in.^8^ An inter-rater reliability test was performed on 20% of the total identified calls, and Cohen’s Kappa revealed a good^45^ level of reliability (K=0.75).

#### Coding and classifying subject behaviours of valence

When they were vocalizing, the subjects’ affective state was classified as either positive, neutral, or negative. We used a combination of facial expressions (in accordance with^1^) and bodily actions (in accordance with^17^). All cues were chosen based on previous studies that found a relationship between that cue and a particular affective state. Affective state cues achieved good levels of inter-rater reliability (K=0.73). For facial expression types, bodily actions, definitions, and associated valence, see Table 2.

If facial expressions and bodily actions matched in their affective valence, then the affective state of the subject was classified as such. If one cue was either positive or negative and the other cue was neutral (e.g. negative facial cue and neutral bodily action), then the affective state of the subject was classified by the former (e.g. negative state). Cases wherein the valence of the cues were contrasting (i.e. positive facial cue and negative bodily action) were not included in the analysis because there was no basis for deciding which affective state should be given priority. Such contrasting cues to affective valence occurred in fewer than 10% of cases. The distribution of facial and bodily cues in realation to vocal production are shown in Tables S1 and S2 respectively.

#### Measuring social partner behaviour

The social partner referred to the individual that the subject was interacting with while vocalising. For infants, this was the mother in 68.37% of cases and another group member in 31.62% of cases. For juveniles, this was the mother in 53.02% of cases and another group member in 46.97% of cases. All mothers were adults. For further details on non-maternal social partner characteristics in relation to their developmental stage and sex, please refer to Tables S3 and S4 respectively. The behaviour of the social partner was coded 4 seconds before the subject’s call, during the call, and 4 seconds after the call. This time frame was chosen because studies that evaluated functional flexibility in the vocal behaviour of human infants also examined caregivers behavioural responses to their infants calls within this time frame.^1^ All observed behaviours were coded using the ethogram developed by.^28^ Social partner behaviour was intra-rater reliability tested using Cohen’s Kappa which showed an excellent degree of reliability was achieved (K = 0.88). See Table 1 below for behaviour categories and the behaviours that comprised each category.

Similar to^1^ we used this data to examine whether social partners continued or changed their behaviour. Continuing behaviour was defined as when the same category of behaviour was observed before, during, and after the subject vocalised. Behavioural change was defined as when a social partner either stopped a behaviour or started showing a new category of behaviour during or after the call was observed.

### Quantification and statistical analysis

#### Criterion 1

To test for the extent to which criterion 1 is satisfied in the vocal repertoire of young chimpanzees (i.e. the degree of affective bias in vocal expression), we first calculated a ‘Berger-Parker diversity index’ value (see^17^^,^^20^) for per call type for each subject. The Berger-Parker diversity index is calculated for each call type by dividing the number of times the call type was produced in the most common affective state by the total number of calls produced across all affective states. Berger-Parker diversity index values range from 1 divided by the number of affective states (in this case 1/3 = 0.33). A value of 0.33 indicates no bias in this case, while a value of 1 indicates complete affective bias. Since a Berger-Parker diversity index value of 1 indicates complete affective bias, we used one-sample Wilcoxon tests for each call type to test whether the observed degree of bias was significantly less than complete affective bias (i.e. a hypothetical median Berger-Parker diversity index value of 1). If a call type satisfies criterion 1, Berger-Parker diversity index values should be significantly lower than 1.

In our analyses, the reader may notice that V=0 for each of our main tests, but the p-values vary. To provide an explanation of this: this statistic measures the sum of positive ranks in the data. A value of 0 can occur for different reasons. One reason is that the number of positive and negative ranks are roughly similar, cancelling each other out, leading to V=0. This is likely the case for the grunts, which contains a mixture of values that would give both positive and negative rank values. This can also stem from there being no difference between the observed value and the hypothetical value of 1 that we are testing our observed values against. For calls that show a strong bias in how they are produced, most of the observed Berger-Parker diversity index values were 1, thus giving rise to a value of V=0. For more information on this topic please refer to.^46^

#### Criterion 2

Among all call types that satisfied criterion 1, we decided to test for criterion 2 (i.e. whether social partners showed consistent behavioural responses to vocalisations depending on how those calls were expressed). To test for criterion 2, we used McNemar tests in order to evaluate whether there was a dependency between the affective state a call expressed (i.e. positive vs negative) and the social partner response (i.e. change vs continue behaviour). If a call type satisfies criterion 2, there should be a significant dependency between how a call is expressed and the type of behavioural response it elicits in a social partner.

All analyses were conducted using base-r functions in the r statistic computing software (V 2023.03.0). Our r-script and all data is available in our supplemental information.

## Data Availability

All data and code have been included as electronic supplementary files with this article.

## Supplemental information

Document S1. Tables S1–S6

Data S1. All data used in analyses, related to Figures 1 and 2

Data S2. All r-code used to produce analysis and figures, related to Figures 1 and 2
