# Supplementary material for: Vocal functional flexibility in the grunts of young chimpanzees
Source: iScience. 2023 Aug 30;26(10):107791. doi: 10.1016/j.isci.2023.107791 (PMC10505970; doi:10.1016/j.isci.2023.107791)
Supplement: Document S1. Tables S1–S6 [file mmc1.pdf]

iScience, Volume *26*

Supplemental information

Vocal functional flexibility in the grunts of young chimpanzees

Derry Taylor, Erik Gustafsson, Guillaume Dezecache, and Marina Davila-Ross

Supplementary Table S1: A table showing the percentage of calls produced with each type of facial expression type observed for each call type, related to the methods subsection 'Coding and classifying subject behaviours of valence', related to table 2.

Table S1. Percentage of each facial expression type observed produced for each vocalisation type

	Vocalisation Type					
**Facial Expression**	Grunt	Hoo	Laugh	Scream	Whimper	**Affect Classification**
Full open-mouth face	24	0	52.80	0	0	Positive
Half open-mouth face	16.23	0	37.70	0	0	Positive
Open-mouth bared-teeth	1.70	0	1.89	68.75	1.96	Negative
Closed-mouth bared-teeth	0.85	0	0	31.25	3.92	Negative
Pout	17.94	67	1.89	0	49.01	Negative
No Expression	39.31	33	5.66	0	45.09	Neutral

Supplementary Table S2: A table showing the percentage of calls produced with each type of bodily behaviour type observed for each call type, related to the methods subsection 'Coding and classifying subject behaviours of valence', related to table 2.

Table S2. Percentage of each category of subject bodily behaviour observed during the production of each call type

	Vocalisation Type					
**Subject Behaviour**	Grunt	Hoo	Laugh	Scream	Whimper	**Affect Classification**
Play	26.96	0	51.8	0	1.36	Positive
Grooming	0	0	0	0	0	Positive
Breastfeeding	0	0	0	0	0.68	Positive
Nuzzling	0	0	0	0	0	Negative
Aggressive actions	0.26	0	0.72	14.63	2.04	Negative
Self-scratching	1.30	0	0	0	0.68	Negative
Avoidance actions	2.09	0	0	0	2.72	Negative
Other actions	69.37	100	85.36	85.36	92.51	Neutral

Supplementary table S3: A table showing the percentage of cases in which the social partner during subject vocalisation was an infant, juvenile, sub-adult, or adult, in relation to subject developmental stage (infant vs juvenile) and sex (male vs female), related to methods subsection 'Measuring social partner behaviour', related to table 3.

Table S3. Developmental stage of social partners during vocal interactions for infant and juvenile males and females. Values are percentages of overall vocal interactions.

	Social Partner Developmental Stage			
**Subject Developmental Stage and Sex**	Infant	Juvenile	Sub-Adult	Adult
Infants (Overall)	19.40	61.23	2.32	17.05
Infant Males	6.82	50	4.54	38.63
Infant Females	25.9	67.05	1.17	5.88
Juvenile (Overall)	3.85	44.50	13.73	37.91
Juvenile Males	2.94	54.41	8.82	33.82
Juvenile Females	6.52	15.21	28.26	50

Supplementary table S4: A table showing the percentage of social partners during subject vocalisation that were male vs female in relation to subject developmental stage (infant vs juvenile) and sex (male vs female), related to methods subsection 'Measuring social partner behaviour', related to table 3.

Table S4. Sex of social partners during vocal interactions for infant and juvenile males and females. Values are percentages of overall vocal interactions

	Social Partner Sex	
**Subject Developmental Stage and Sex**	Male	Female
Infant (Overall)	64.34	35.65
Infant Male	88.63	11.36
Infant Female	51.76	48.23
Juvenile (Overall)	77.59	22.40
Juvenile Male	81.02	18.98
Juvenile Female	67.39	32.60

Supplementary table S5: A table showing the raw number of social partner behavioural responses (change vs continue) in response to combinations of subject behaviour and facial expression during the production of grunt vocalisations, related to results subsection 'Criterion 2', related to table 2 and table 3.

Table S5. Subject behaviour, facial expression, and behavioural response of mothers during grunt production. Values refer to number of observer cases.

**Subject behaviour**	**Subject Facial Expression**	**Behavioural response**	
		Change	Continue
Play	Full open-mouth face	10	12
Play	Half open-mouth face	9	3
Play	Open-mouth bared-teeth	1	0

Play	Closed-mouth bared-teeth	1	0
Play	Relaxed face	4	3
Play	Pout	1	0
Aggressive	Full open-mouth face	1	0
Aggressive	Half open-mouth face	1	0
Aggressive	Relaxed face	1	0
Self-scratching	Relaxed face	2	0

Supplementary table S6: A table showing the raw number of social partner behavioural responses (change vs continue) in relation to the subjects behaviour, subjects facial expression, social partner sex (male vs female) and social partner developmental stage (infant, juvenile, sub-adult, and adult), related to results subsection 'Criterion 2', related to table 2 and table 3.

Table S6. Subject behaviour, facial expression, social partner characteristics (sex and developmental stage) and social partner behaviour responses during grunt production. Values refer to number of observed cases

**Social Partner Sex**	**Social Partner Stage**	**Subject Behaviour**	**Subject Facial Expression**	**Behavioural Response**	
				Change	Continue
Female	Adult	Play	Full open-mouth face	1	1
Female	Adult	Play	Half open-mouth face	0	1
Female	Sub-adult	Play	Full open-mouth face	1	0
Female	Sub-adult	Play	Relaxed face	0	1
Female	Juvenile	Play	Full open-mouth face	1	0
Female	Juvenile	Play	Half open-mouth face	2	0
Female	Juvenile	Play	Relaxed face	1	1
Female	Juvenile	Play	Pout	1	0
Female	Infant	Play	Full open-mouth face	0	1
Male	Adult	Play	Full open-mouth face	0	1
Male	Adult	Play	Half open-mouth face	1	0
Male	Sub-adult	Play	Half open-mouth face	1	0
Male	Juvenile	Play	Full open-mouth face	0	7

Male	Juvenile	Play	Half open-mouth face	3	2
Male	Infant	Play	Full open-mouth face	0	1
Male	Infant	Play	Half open-mouth face	1	1
Male	Infant	Self-scratch	Relaxed face	1	0
Female	Juvenile	Aggressive	Relaxed face	1	0
Female	Juvenile	Aggressive	Closed-mouth bared-teeth	1	0
Female	Juvenile	Aggressive	Half open-mouth face	1	0
Female	Juvenile	Aggressive	Full open-mouth face	1	0
Male	Juvenile	Aggressive	Closed-mouth bared-teeth	1	0
Male	Juvenile	Aggressive	Half open-mouth face	1	0
Female	Adult	Avoidance	Relaxed face	1	0
Female	Sub-adult	Avoidance	Relaxed face	1	0
Female	Sub-adult	Avoidance	Half open-mouth face	1	0
Male	Adult	Avoidance	Full open-mouth face	1	0
Male	Adult	Avoidance	Pout	2	0
Male	Adult	Avoidance	Relaxed face	1	1
Male	Sub-adult	Avoidance	Pout	1	0
Male	Juvenile	Avoidance	Half open-mouth face	0	1
Male	Juvenile	Avoidance	Relaxed face	1	1
Male	Infant	Avoidance	Half open-mouth face	1	0
